# Dynamic Reprogramming of DNA Methylation at an Epigenetically Sensitive Allele in Mice

**DOI:** 10.1371/journal.pgen.0020049

**Published:** 2006-04-07

**Authors:** Marnie E Blewitt, Nicola K Vickaryous, Andras Paldi, Haruhiko Koseki, Emma Whitelaw

**Affiliations:** 1 School of Molecular and Microbial Biosciences, University of Sydney, New South Wales, Australia; 2 Genethon and EPHE, Evry, France; 3 Developmental Genetics Group, RIKEN Research Center for Allergy and Immunology, Yokohama, Japan; 4 Queensland Institute of Medical Research, Brisbane, Queensland, Australia; Stanford University School of Medicine, United States of America

## Abstract

There is increasing evidence in both plants and animals that epigenetic marks are not always cleared between generations. Incomplete erasure at genes associated with a measurable phenotype results in unusual patterns of inheritance from one generation to the next, termed transgenerational epigenetic inheritance. The *Agouti viable yellow (A^vy^)* allele is the best-studied example of this phenomenon in mice. The *A^vy^* allele is the result of a retrotransposon insertion upstream of the *Agouti* gene. Expression at this locus is controlled by the long terminal repeat (LTR) of the retrotransposon, and expression results in a yellow coat and correlates with hypomethylation of the LTR. Isogenic mice display variable expressivity, resulting in mice with a range of coat colours, from yellow through to agouti. Agouti mice have a methylated LTR. The locus displays epigenetic inheritance following maternal but not paternal transmission; yellow mothers produce more yellow offspring than agouti mothers. We have analysed the DNA methylation in mature gametes, zygotes, and blastocysts and found that the paternally and maternally inherited alleles are treated differently. The paternally inherited allele is demethylated rapidly, and the maternal allele is demethylated more slowly, in a manner similar to that of nonimprinted single-copy genes. Interestingly, following maternal transmission of the allele, there is no DNA methylation in the blastocyst, suggesting that DNA methylation is not the inherited mark. We have independent support for this conclusion from studies that do not involve direct analysis of DNA methylation. Haplo-insufficiency for Mel18, a polycomb group protein, introduces epigenetic inheritance at a paternally derived *A^vy^* allele, and the pedigrees reveal that this occurs after zygotic genome activation and, therefore, despite the rapid demethylation of the locus.

## Synopsis

There is now a reasonable amount of evidence from both epidemiological studies in humans and from genetic studies in animals and plants that information in addition to the primary DNA sequence is inherited across generations and can influence the phenotype of the offspring. Researchers refer to this information as epigenetic, and there is much interest in discovering the molecular basis for this epigenetic information. They now know a great deal about the various types of epigenetic marks that regulate the expression of the genome within the life of an organism, and these include both modifications to the DNA molecule itself, specifically DNA methylation and modifications to the proteins that package the DNA into chromosomes, termed chromatin. DNA methylation appears to be one of the most stable epigenetic modifications and has been the primary candidate for the molecule responsible for transgenerational epigenetic inheritance. The results presented here suggest that DNA methylation is not the inherited epigenetic mark, at least in the mouse model used in this study.

## Introduction

Epigenetic reprogramming of the genome is an essential process that occurs during both primordial germ cell development and early embryogenesis so that the epigenetic marks are cleared and reset between generations [1]. Occasionally, it appears that this epigenetic reprogramming is incomplete, since we observe the inheritance of epigenetic state, termed transgenerational epigenetic inheritance [2]. The molecular nature of the epigenetic mark that is inherited is not known. Non-Mendelian inheritance, such as this, is of particular interest if it exists in humans, since it would have a profound effect on the inheritance of phenotypic traits.

**Figure 1:**
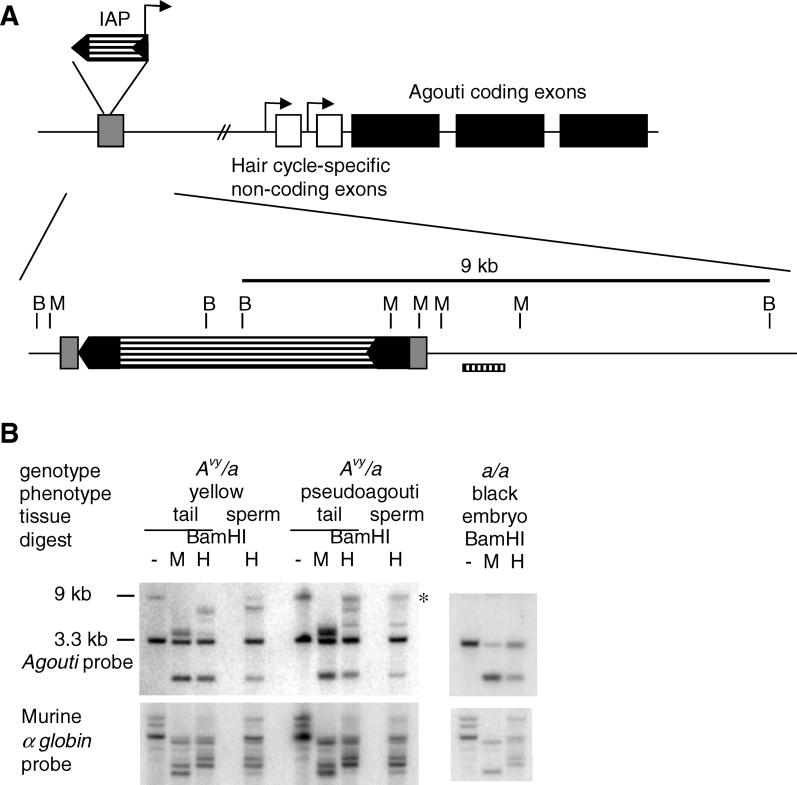
Methylation of the *A^vy^* Allele in Mature Sperm (A) Expression of the *A^vy^* allele is controlled by an IAP, inserted into pseudoexon 1a (grey box). A cryptic promoter within the 3′ LTR of the IAP (black arrows) directs transcription of the *agouti* coding exons. The BamHI (B) and MspI (M) sites are shown in the region of the unique 400-bp probe B. Tail and mature sperm from a yellow and a pseudoagouti male were collected. DNA was prepared and samples digested with BamHI followed by MspI or its isoschizomer HpaII, transferred and hybridised with the *agouti* probe [3]. The *A^vy^* allele produces a 9-kb BamHI band, while the *a* allele produces a 3.3-kb band. Membranes were stripped and rehybridised with a murine *α-globin* probe to check for equal digestion within the tissue samples (shown in [B]). These results represent experiments performed on sperm and tail DNA from seven yellow and five pseudoagouti males, a further one of each are shown in Figure S1. Mature sperm were isolated from both epididymes of the male (each sample contained in the order of 10^6^ to 10^7^ spermatocytes). Sperm samples were checked by light microscopy and found to be greater than 95% spermatocytes. The methylation state of the tissues is indicated by the ratio of the 9-kb BamHI band to the 7-kb band remaining after HpaII digestion. The 9-kb band is marked by an asterisk. The methylation state of the sperm reflects the phenotype of the father rather than the range of phenotypes seen in the offspring.

Arguably the best characterised instance of transgenerational epigenetic inheritance in mammals occurs following female transmission of the *Agouti viable yellow (A^vy^)* allele in mice [3]. Expression of the *A^vy^* allele is controlled by an intracisternal A particle (IAP), inserted into pseudoexon 1a of the *Agouti* gene (Figure 1A). A cryptic promoter within the 3′ long terminal repeat (LTR) of the IAP directs transcription of the *Agouti* coding exons. The activity of the *A^vy^* allele is variable among isogenic littermates and correlates with epigenetic state; the silent allele is hypermethylated and produces a wild-type agouti-coloured coat (termed pseudoagouti), while the active allele is hypomethylated and produces a completely yellow coat [3]. The activity of the allele can differ between cells, termed variegation, producing a mottled mouse, with patches of yellow and patches of pseudoagouti fur. The locus displays epigenetic inheritance following maternal transmission; yellow mothers produce more yellow offspring than pseudoagouti mothers. Interestingly, the *A^vy^* allele does not display epigenetic inheritance following paternal transmission: yellow and pseudoagouti *A^vy^/a* sires produce the same proportion of phenotypes in their offspring. Why epigenetic inheritance occurs following maternal, but not paternal, transmission of the allele is not understood, but we have evidence that this is a strain background effect since transgenerational epigenetic inheritance does occur following paternal transmission of the Axin fused allele in the 129 background [4]. Our hypothesis is that transgenerational epigenetic inheritance is the result of a failure to clear epigenetic marks.

It has been reported that IAPs in general appear to be resistant to epigenetic reprogramming [5]. We were interested in learning more about both the molecular nature of this epigenetic inheritance and the stage, during gametogenesis and early embryonic development, at which the epigenetic state is reprogrammed. To this end, we have analysed the DNA methylation state of the *A^vy^* allele in gametes, zygotes, and blastocysts, following both paternal and maternal transmission. This is the first report of bisulfite sequencing analysis on a nonimprinted allele, followed separately through maternal and paternal inheritance. We found that DNA methylation at the LTR of the IAP at the *A^vy^* allele is reprogrammed in early development in a similar way to the maternal and paternal genomes in general. Furthermore, we found a complete clearing of DNA methylation following maternal transmission, which argues against DNA methylation being the meiotically inherited epigenetic mark. In addition, we provide independent evidence from genetic studies, using a polycomb group knockout mouse, to support this conclusion.

## Results

### A Paternally Derived *A^vy^* Allele Is Subject to Rapid Demethylation Immediately Postfertilisation

**Figure 2:**
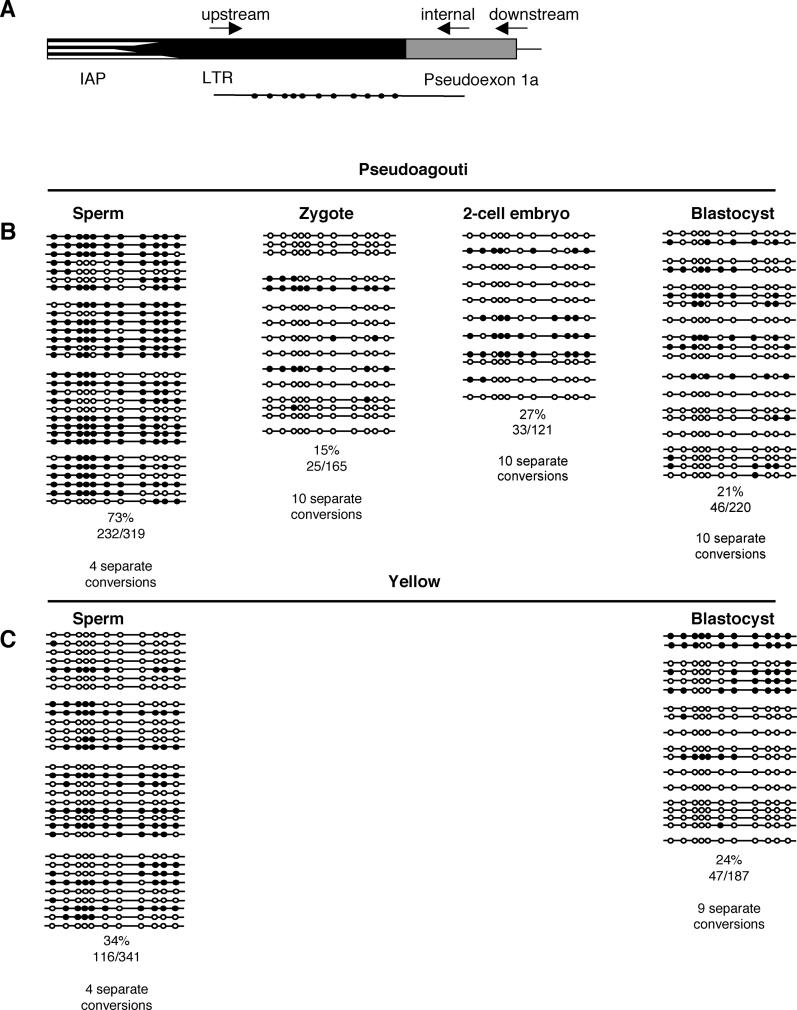
Methylation of the *A^vy^* Allele following Paternal Transmission The methylation status of each CpG dinucleotide was determined by sequencing PCR clones of bisulfite-converted DNA [4]. Each line represents an individual clone, theoretically from one cell, and each circle an individual CpG. Open circles indicate an unmethylated CpG, and closed circles a methylated CpG. Each block of lines represents the clones derived from the sperm of one adult male mouse, one set of ten zygotes, ten two-cell embryos or one blastocyst. The percentage of methylation in each dataset is shown (calculated from the number of methylated CpGs divided by the total CpGs sequenced, multiplied by 100). The position of each circle is representative of the relative location along the length of the PCR product. Any clones with greater than 5% non-CpG methylation were excluded from the dataset, and these clones made up less than 5% of all clones sequenced. These clones tended to have very high levels of non-CpG methylation, an indication of incomplete bisulfite conversion. Zygotes, two-cell embryos, or blastocysts were collected from yellow or pseudoagouti *A^vy^/a* sires mated to *a/a* dams, as indicated. Clones were only included in the zygote or blastocyst samples if they could be distinguished from others in the sample by CpG or low-level non-CpG methylation. (A) The IAP LTR pseudoexon 1a junction shown in detail. The bisulfite sequencing primers are shown [4]. The PCR product contains 11 CpG dinucleotides, depicted as circles, all of which are in the LTR. (B) Data obtained from the sperm of four pseudoagouti males, and 10 × 10 zygotes, 10 × 10 two-cell embryos or 10 × 1 blastocysts collected from pseudoagouti *A^vy^/a* sires mated to *a/a* dams. (C) Data obtained from the sperm of four yellow male and 9 × 1 blastocysts collected from yellow *A^vy^/a* sires mated to *a/a* dams. These data indicate that the *A^vy^* allele is subject to rapid demethylation immediately postfertilisation following paternal transmission.

The DNA methylation state at the *A^vy^* allele in sperm of yellow and pseudoagouti males was determined by Southern transfer and by bisulfite sequencing. Tail and sperm DNA from yellow and pseudoagouti males were digested with BamHI, followed by MspI or HpaII. MspI and HpaII are isoschizomers that recognise the sequence CCGG. HpaII does not cut when the internal cytosine is methylated, whereas MspI cuts irrespective of methylation at this internal cytosine residue. The samples were analysed by Southern transfer, using the *agouti* probe (see Figure 1A). Each mouse carries both the *A^vy^* and *a* alleles. The *a* allele is a null allele found in the C57BL/6 background. Following BamHI digestion, the *agouti* probe detects a 9-kb and 3.3-kb band from the *A^vy^* and *a* alleles, respectively [3]. As previously shown [3], the 9-kb BamHI band, representing the *A^vy^* allele, is reduced in size following HpaII digestion of DNA from the tails of yellow mice, whereas a significant amount of the band remains uncut in the DNA from the tails of pseudoagouti mice (Figure 1B, upper panel; Figure S1), indicating a higher degree of methylation in tail tissue of a pseudoagouti animal. Similarly, the sperm of yellow mice are less methylated than those of pseudoagouti mice (Figure 1B and Figure S1). This result confirms our bisulfite sequencing analysis of sperm DNA (Figure 2A–2C), which has been published previously [4]. The *A^vy^* allele has an average of 73% methylation in sperm of pseudoagouti males, compared with 34% in the sperm of yellow males. In this genetic background (C57BL/6), the range of phenotypes of the offspring of yellow and pseudoagouti males is the same [3]. So the methylation of the *A^vy^* allele in sperm reflects the phenotype of the father, rather than the range of phenotypes seen in his offspring. Therefore, at the paternally inherited allele, it appears that the epigenetic mark must be cleared and reestablished after fertilisation [4].

We went on to perform bisulfite sequencing on zygotes, two-cell embryos, and blastocysts produced from pseudoagouti *A^vy^/a* sires (Figure 2B and 2C). We used a rigorous method to avoid clonal bias: within one single bisulfite conversion, clones were only included in the dataset if they could be distinguished as different by either CpG or non-CpG methylation. The bisulfite conversions were carried out on multiple separate occasions for each time point. The zygotes show a dramatic and highly significant (*p* < 0.001, one-tailed t-test, equal variance) decrease in methylation from the level seen in mature sperm (Figure 2B). We analysed zygotes 21–23 h after the administration of human chorionic gonadotrophin, corresponding to 9–11 h postfertilisation. DNA replication is not complete until 12 to 14 h postfertilisation [6,7]. Therefore the drop from 73% to 15% methylation that occurred in less than 11 h, and before the first cell division, is likely to be due to active demethylation. In this respect, the *A^vy^* allele is behaving like the paternal genome in general [8,9], rather than like the IAPs in general [5]. The IAP at *A^vy^* is unusual since it can be active, unlike the vast majority of IAP LTRs, which remain heavily methylated and presumably transcriptionally inactive.

The level of methylation remained low in the two-cell embryos and blastocysts (Figure 2B and 2C). The blastocysts from yellow and pseudoagouti sires had equivalent levels of methylation, in contrast to the significant difference in methylation in the sperm of these males. This suggests that the clearing of methylation marks on the paternally derived allele is basically complete in the zygote. The level of methylation in blastocysts appears to be much lower than that of somatic tissue, so presumably resetting has not yet occurred.

**Figure 3:**
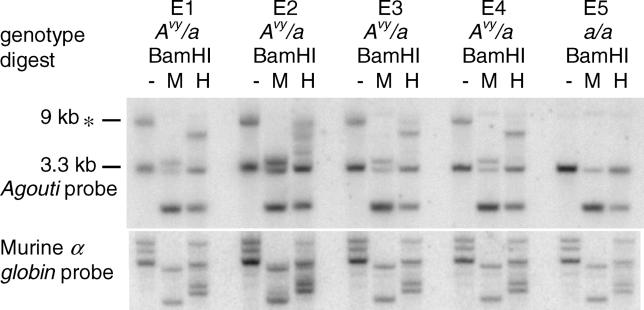
Methylation of the *A^vy^* Allele in 12.5-dpc Embryos following Paternal Transmission The 12.5-dpc embryos produced from an *A^vy^/a* sire mated with an *a/a* dam. Samples were digested and subjected to Southern transfer as described in Figure 1. A range of methylation states were observed, evidenced by the varying amounts of the 9-kb BamHI band remaining after HpaII digestion. This indicates that the methylation is likely to be reset by this stage of development.

By 12.5 days postcoitum (dpc), a full range of methylation states are observed in embryos produced from *A^vy^/a* sires (Figure 3 and Figure S2): E1 and E4 are hypomethylated, E2 is hypermethylated, and E3 is moderately methylated, as indicated by the variable amount of the 9-kb BamHI band remaining after HpaII digestion (Figure 3). These methylation states are similar to those seen in somatic tissue of yellow, pseudoagouti, and mottled adults (Figure 1B and [3]). This has been confirmed by bisulfite sequencing (Figure S2). A similar result is seen at 10.5 dpc (unpublished data). Therefore, at the paternally inherited *A^vy^* allele, it appears that the epigenetic mark is actively cleared immediately postfertilisation and reestablished in a stochastic manner before midgestation. Resetting of the epigenetic marks at the locus is likely to occur at approximately the same stage as X inactivation (6.5 dpc) [10,11]; however, given the time points we have analysed, our data only allow us to place the reestablishment of the epigenetic marks between implantation and midgestation.

### A Maternally Inherited *A^vy^* Allele Is Not Subject to Rapid Demethylation, but Is Completely Demethylated before Implantation

**Figure 4:**
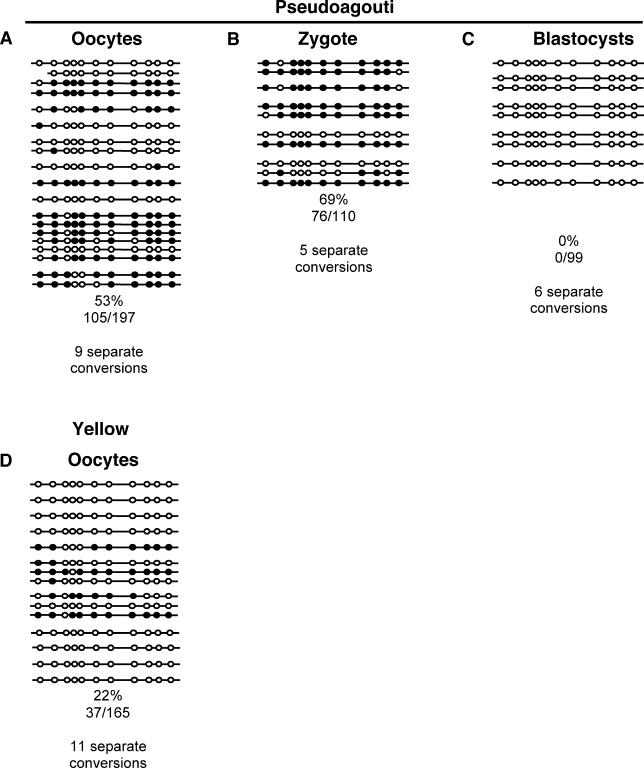
Methylation of the *A^vy^* Allele following Maternal Transmission The methylation status of each CpG dinucleotide was determined by sequencing PCR clones of bisulfite-converted DNA, as described in Figure 2 [4]. Each block of lines represents the clones derived from one bisulfite conversion of ten cells (oocytes or zygotes). The percentage of methylation in each dataset is shown (calculated from the number of methylated CpGs divided by the total CpGs sequenced, multiplied by 100). Clones were only included in the samples if they could be distinguished from others in the sample by CpG or non-CpG methylation. Any clones with higher than 5% non-CpG methylation (an indication of incomplete bisulfite conversion) were excluded from the dataset, and these clones made up less than 5% of all clones sequenced. (A) and (D) Unfertilised oocytes from pseudoagouti or yellow *A^vy^/a* females, respectively. DNA methylation at the *A^vy^* allele does not appear to have been reset during oogenesis. (B) Zygotes from pseudoagouti *A^vy^/a* dams mated to *a/a* sires. (C) Blastocysts from pseudoagouti *A^vy^/a* dams mated to *a/a* sires. The *A^vy^* allele is not subject to rapid demethylation immediately postfertilisation following maternal inheritance, but is completely cleared of DNA methylation before blastocyst formation.

We performed bisulfite sequencing on unfertilised oocytes from yellow and pseudoagouti females, and zygotes and blastocysts from pseudoagouti females (Figure 4). As expected, the allele was more heavily methylated in oocytes of pseudoagouti females than in those of yellow females (Figure 4A and 4D). Moreover, the level of methylation in the zygotes of pseudoagouti females was similar to that found in the unfertilised oocytes (Figure 4A and 4B). There is no rapid demethylation of the maternally transmitted *A^vy^* allele in the zygote. This raises the possibility that the absence of rapid demethylation of the maternally transmitted allele contributes to the transgenerational epigenetic inheritance. However, blastocysts from pseudoagouti dams did not show any methylation at the allele (Figure 4C). Pseudoagouti dams produce 20% pseudoagouti offspring, whereas yellow dams do not produce any [3]. These results suggest that DNA methylation is not the inherited mark at the *A^vy^* allele, as it is inconsistent with the requirement for incomplete erasure following transmission of the allele from a pseudoagouti dam.

### Transgenerational Epigenetic Inheritance at the *A^vy^* Allele Is Seen following Paternal Transmission in Offspring That Are Haplo-Insufficient for Mel18

**Figure 5:**
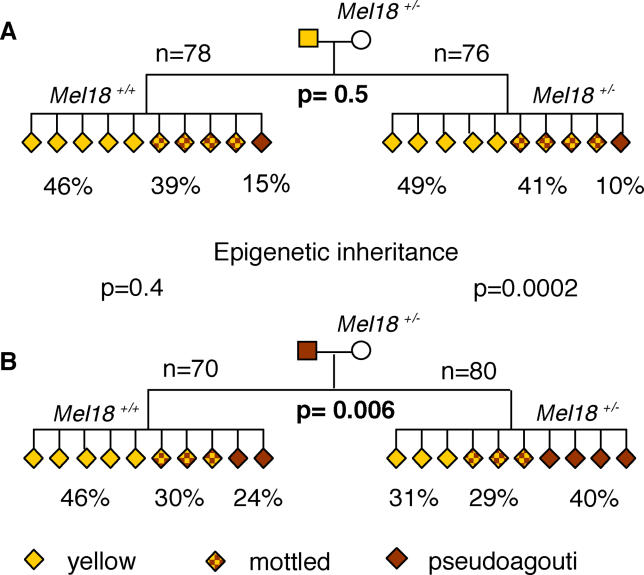
Pedigrees of Crosses between *Mel18^+/−^* and Mice Carrying the *A^vy^* Allele *A^vy^/a* C57BL/6J sires of the indicated phenotype were mated with *Mel18^+/−^* C57BL/6J dams. Data were produced from at least five different mating pairs in each case. Offspring not carrying the *A^vy^* allele have been omitted. All coat colour phenotypes were scored by one observer, before the analysis of *Mel18* genotype. (A) There was no significant shift in the proportion of phenotypes observed between *Mel18^+/−^* and *Mel18^+/+^* littermates from yellow sires. (B) There was a significant shift towards the pseudoagouti phenotype in *Mel18^+/−^* compared with *Mel18^+/+^* littermates from pseudoagouti sires (*p* = 0.006). This shift produced a statistically significant difference in the range of *Mel18^+/−^* offspring observed from yellow and pseudoagouti sires (*p* = 0.0002); the *Mel18^+/−^* offspring of pseudoagouti sires are more likely to be pseudoagouti than those of yellow sires, i.e., transgenerational epigenetic inheritance is observed. Epigenetic inheritance was not observed in the *Mel18^+/+^* littermates.

We wished to find independent evidence that DNA methylation may not be the inherited mark at the *A^vy^* allele. In *Drosophila,* polycomb group proteins have been shown to be involved in transgenerational epigenetic inheritance [12,13]. For this reason, we tested the effect of haplo-insufficiency for Mel18 (also known as Rnf110 or Pcgf2), a mammalian polycomb group protein [14], on epigenetic inheritance at the *A^vy^* allele. We mated *Mel18^+/−^* mice with mice carrying the *A^vy^* allele. When the *A^vy^* allele was maternally inherited, and the *Mel18* knockout allele paternally inherited, epigenetic inheritance was observed in both wild-type and *Mel18^+/−^* offspring (unpublished data). Interestingly, when the *A^vy^* allele was paternally inherited (and the *Mel18* knockout allele maternally inherited), epigenetic inheritance was observed (Figure 5A and 5B). The *Mel18^+/−^* offspring of pseudoagouti sires are more likely to be pseudoagouti than those of yellow sires (*p* = 0.0002). Epigenetic inheritance is not usually observed in this genetic background (C57BL/6) following paternal transmission of the *A^vy^* allele [3]. Consistent with this previous finding, we observe epigenetic inheritance in the mice haplo-insufficient for Mel18, but not in their wild-type littermates. The cytoplasmic environment of oocytes is established before the segregation of the diploid set of chromosomes. Therefore, the fact that this effect is not seen in the wild-type littermates argues that it is dependent on activation of the zygotic genome and, hence, events after zygotic genome activation at the two-cell stage [15]. Active demethylation of the paternal genome occurs in the first 6 h postfertilisation ([9] and Figure 2), so is complete by the time the wild-type and *Mel18* knockout embryos differ in their Mel18 complement. These results are consistent with the idea that DNA methylation is not the inherited mark.

## Discussion

Transgenerational epigenetic inheritance has been a controversial phenomenon since it was first reported in plants [16]. Since this time, it has been observed in yeast, *Drosophila,* and mammals at both transgenes and endogenous alleles [17]. We and others have suggested that epigenetic inheritance may result from the incomplete clearing of epigenetic marks during either primordial germ cell development or early embryonic development [2]. The *A^vy^* allele in mice has become the principal model system to study epigenetic inheritance, and recently, the *A^vy^* allele has been used to study the effect of maternal diet on phenotype [18,19]. To enable a clearer interpretation of all the current studies, we need to understand the molecular basis of the epigenetic reprogramming at the *A^vy^* allele.

We have found that DNA methylation at the *A^vy^* allele is not reprogrammed during primordial germ cell development. However, during preimplantation development, the paternal allele is rapidly demethylated immediately following fertilisation, whereas the maternal allele is not. By blastocyst stage, the maternal allele is completely demethylated, presumably via a passive mechanism. These results are in contrast to the behaviour of the majority of IAPs, which are largely resistant to reprogramming [5], but are consistent with the behaviour of maternal and paternal genomes in general [8,9]. Epigenetic inheritance at this locus in this genetic background occurs following maternal transmission of the *A^vy^* allele. If this were the result of incomplete clearing of DNA methylation at the allele, then there would be a requirement for some DNA methylation to remain on the maternally transmitted allele at the blastocyst stage, which we do not see. These results suggest that DNA methylation is not the inherited epigenetic mark.

We have presented independent evidence for this conclusion. We found that following paternal transmission of the *A^vy^* allele, and maternal transmission of the *Mel18* knockout allele, mice haplo-insufficient for Mel18 displayed epigenetic inheritance, whereas their wild-type littermates did not. This implicates events after zygotic genome activation, and therefore after the complete demethylation of the *A^vy^* allele, supporting our conclusion that DNA methylation does not appear to be the inherited epigenetic mark at this allele. It is interesting that in a *Mel18* haplo-insufficient background, there is transgenerational epigenetic inheritance, whereas in a *Mel18* wild-type background this is not the case. Presumably, some underlying epigenetic mark at *A^vy^* is cleared in *Mel18* wild-type embryos but not fully cleared in *Mel18* haplo-insufficient embryos. However, the role played by *Mel18* in the clearing of this mark is not fully understood. Normally, Mel18 is part of polycomb repressive complex 1, which performs histone H2A ubiquitination, interacts with histone methyltransferases, histones, and chromatin remodelling complexes [20–22]. Polycomb complexes are dynamic in their composition [23], and reducing the level of Mel18 may have altered the makeup of PRC1, in turn affecting epigenetic reprogramming.

It has been known for some time that epigenetic marks can be inherited during meiosis in fission yeast, which do not methylate their DNA at all [24]. Furthermore, in a study of the inheritance of DNA methylation patterns in humans, Silva and White found that although the level of DNA methylation was heritable in families, the methylation patterns were not identical across generations or between tissues [25]. They concluded that DNA methylation was a secondary mark, rather than the primary inherited epigenetic mark.

If DNA methylation is not the heritable mark that produces transgenerational epigenetic inheritance, it seems likely that specific histone modifications play a role. In this regard, it is interesting that histone H4 lysine 20 trimethylation has been reported in embryonic stem cells as the major histone methylation mark of IAP LTRs [26]. Alternatively, there may be an RNA-based mechanism that accounts for the inheritance. Unfortunately, no methods are currently available to study either chromatin state at a specific locus or small RNA molecules with sufficient sensitivity to analyse these marks at the *A^vy^* allele during the very early stages of development.

Epigenetic inheritance is a controversial phenomenon, which fundamentally changes the way that we interpret inheritance of phenotypic traits. Although it is not yet clear whether it occurs in humans, epigenetic inheritance has now been reported to occur at a number of alleles in mice [17], providing a tractable system in which to study this event. We have found that at the *A^vy^* allele, DNA methylation is unlikely to be the epigenetic mark that is inherited from one generation to the next.

## Materials and Methods

### Methylation-sensitive restriction enzyme digestion and Southern transfer.

DNA was prepared from tail, sperm, or embryo and digested and subjected to Southern transfer as previously described [3,4]. The membrane was stripped of the *Agouti* probe and hybridised with a probe to the mouse *α-globin* locus to control for equal digestion [3].

### Animal husbandry and embryo dissection.

Both the *A^vy^* allele and the *Mel18* knockout allele are maintained on the C57BL/6 inbred strain so that pseudoagouti *A^vy^/a* animals are visibly different to wild-type *a/a* animals.

For dissection of oocytes, zygotes, two-cell embryos, or blastocysts, virgin females were superovulated. The females were injected intraperitoneally with 10 U of pregnant mare serum gonadotrophin (Folligon) on day 1 between hours 8 and 9 of a 12-h day–night cycle. On day 3 (47 h later) they were injected with 10 U of chorionic gonadotrophin (Chorulon) between hours 7 and 8 of a 12-h day–night cycle. For oocytes, the females were dissected 21–23 h after the administration of Chorulon. For zygotes, two-cell, or blastocysts, the superovulated females were set up with males and dissected 21–23 h, 45–47 h, or 93–95 h postadministration of Chorulon for the females where a vaginal plug was detected.

For dissection of 12.5- and 10.5-dpc embryos, *a/a* females were mated with pseudoagouti *A^vy^/a* males. The day of detection of a vaginal plug was counted as day 0.5.

### Bisulfite treatment and sequencing.

Bisulfite conversion of DNA was performed as previously described [4], except that ten oocytes, ten zygotes, ten two-cell embryos, or one blastocyst were embedded directly in agarose. Each sample was incubated overnight in 2 mg/ml proteinase K, 0.5 M EDTA. After bisulfite conversion, each agarose block was resuspended in 10 μL of water, and 5 μL was used in the primary PCR. An agarose-only control was always included, and the experiment was only used if the agarose control was negative at the end of the seminested PCR. PCR fragments were subcloned into pGEM-T Easy (Promega, Madison, Wisconsin, United States). Clones were only accepted if they differed in CpG methylation, or non-CpG methylation. Any clones with greater than 5% non-CpG methylation were excluded from the dataset, although these made up less than 5% of all clones sequenced. These clones tended to have very high levels of non-CpG methylation, an indication of incomplete bisulfite conversion. For the early embryos, in general, only one clone was included per sample, since they were largely unmethylated, meaning our calculation of methylation is likely be an overestimate and is therefore a conservative estimate of demethylation.

### Genotyping Mel18 knockout mice.

Tail DNA was prepared as previously described [3]. The *Mel18* knockout allele was detected using primers specific for the *Neo* cassette, in the same PCR reaction as a control *Tcrd* primer pair, as described for *Dnmt1* and other *Neo* knockouts of the Jackson Laboratory (http://www.jax.org).

## Supporting Information

Figure S1 Methylation of the *A^vy^* Allele in Mature Sperm Tail and mature sperm from one yellow and one pseudoagouti male were collected. Samples were digested and subjected to Southern transfer as described in Figure 1. The difference in methylation state between the sperm of yellow and pseudoagouti males is evidenced by the varying amounts of the 9-kb BamHI band remaining after HpaII digestion (asterisk). The methylation state of the sperm reflects the phenotype of the father rather than the range of phenotypes seen in the offspring.
(87 KB DOC)

Figure S2 Bisulfite Analysis of Methylation of the *A^vy^* Allele in 12.5-dpc Embryos The 12.5 dpc embryos were produced from an *A^vy^/a* sire mated with an *a/a* dam (paternal transmission), or an *a/a* sire mated with an *A^vy^/a* dam. The methylation status of each CpG dinucleotide was determined by sequencing PCR clones of bisulfite-converted DNA, as described in Figure 2. Each block of lines represents the clones derived from one bisulfite conversion of one 12.5-dpc embryo. Any clones with higher than 5% non-CpG methylation (an indication of incomplete bisulfite conversion) were excluded from the dataset, and these clones made up less than 5% of all clones sequenced. The embryos were chosen for bisulfite conversion after they were first analysed using methylation-sensitive restriction enzymes and Southern transfer, as shown in Figure 3. Embryos were chosen that appeared hypermethylated (A) or hypomethylated (B) on the Southern transfer, and bisulfite sequencing was performed to validate the Southern transfer results. The percentage of methylation in the hypomethylated or hypermethylated class is shown (calculated from the number of methylated CpGs divided by the total CpGs sequenced, multiplied by 100). At 12.5 dpc, we observed embryos that were hypermethylated at the *A^vy^* allele, as well as embryos that were hypomethylated, indicating that the methylation is reset by this stage of development.
(195 KB DOC)

### Accession Numbers

The Entrez Gene (http://www.ncbi.nlm.nih.gov/entrez/query.fcgi?db=gene) accession number for Mel18 is 22658 (also known as Rnf110 or Pcgf2).

## Abbreviations

*A^vy^*: *Agouti viable yellow*
dpc: days postcoitum
IAP: intracisternal A particle
LTR: long terminal repeat
