# Supplementary material for: Dynamic Reprogramming of DNA Methylation at an Epigenetically Sensitive Allele in Mice
Source: PLoS Genet. 2006 Apr 7;2(4):e49. doi: 10.1371/journal.pgen.0020049 (PMC1428789; doi:10.1371/journal.pgen.0020049)
Supplement: Supplementary file 1 [file pgen.0020049.sg001.doc]

genotype

phenotype

tissue

digest

*A^vy^/a*

yellow

tail	sperm

BamHI

- M H	H

*A^vy^/a*

pseudoagouti

tail	sperm

BamHI

- M H	H

9 kb

3.3 kb

*Agouti* probe

Murine *α* *globin* probe

*
