# Supplementary material for: Dynamic Reprogramming of DNA Methylation at an Epigenetically Sensitive Allele in Mice
Source: PLoS Genet. 2006 Apr 7;2(4):e49. doi: 10.1371/journal.pgen.0020049 (PMC1428789; doi:10.1371/journal.pgen.0020049)
Supplement: Supplementary file 2 [file pgen.0020049.sg002.doc]

**A**

**Male transmission**

**Female transmission**

84%

147/176

**Hypermethylated**

**Hypomethylated**

# B

**Male transmission**

**Female transmission**

9.7 kb

3.5 kb

*Agouti* probe

Murine *α* *globin* probe

17%

33/198
